# Quackery in Medical Journalism

**Published:** 1872-03

**Authors:** 


QUACKERY IN MEDICAL JOURNALISM.—"Several American medical journals continue the disgusting business of publishing commendatory letters sent to them. This charlatanism cannot be sufficiently rebuked. One almost imagines, when reading such matter, that he has, by mistake, taken up the almanac of some nostrum vendor. Commendatory letters are very grateful and welcome to all editors, but they are certainly never sent to subserve the purpose of advertisements." *Richmond and Louisville Medical Journal.*

DR. JNO. STAINBACK WILSON requests the discontinuance of his card, published in the last number of the JOURNAL, soliciting contributions of books, etc., in behalf of the Atlanta Academy of Medicine.

